# Reviews and Book Notices

**Published:** 1880-01

**Authors:** 


### Reviews and Book Notices.

ARTICLE IX.—HYGIENE AND PUBLIC HEALTH. Edited by Albert H. Buck, M.D. New York: William Wood & Co. Two volumes. Octavo.

[Concluded from page 655, Vol. XXXIX, No. 6.]

The second volume is opened by a lengthy paper by Dr. Roger S. Tracy, of New York, on the "Hygiene of Occupation." This treats of the various diseases which are produced by different trades and occupations. Among the diseases produced by chemicals is noticed a curious eczema which is caused by sulphate of quinine. In the great factory of Powers & Weightman this eruption occurs in about 90 per cent. of the new workmen, three months after entrance. "The majority are only affected once, while others have two or three attacks. Some cannot become accustomed to it. It is exceedingly rare for any one to have it if working in the dry materials. The vapor from boiling solutions seems to be the chief cause." The bearing of this upon the mode of action of vegetable organic matters that are highly nitrogenized, and operating in connection with heat and moisture, hardly needs comment.

Our author finds that "fat-renderers, lard-refiners, bone-boilers, glue-makers, fertilizer-makers, pork-packers, soap-makers, oil-pressers and cheese-makers are exposed to offensive gases, but, so far from suffering in health on that account, appear to be actually benefited." Scavengers, also, "are in general a healthy class of men;" all of which corresponds exactly with our experience in Chicago. Physicians are credited with a very high rate of mortality. "In England the average age of physicians at death is given as 45. Old physicians are rare."

The Hygiene of Camps forms the subject of a valuable paper by Assist-Surgeon Charles Smart, U. S. A., an author who has written an interesting essay on "Malarious Waters and Mountain Fever," published in the *American Journal of Medical Sciences*, Jan., 1878. He naturally recurs to the same line of thought when treating of the water-supply and diseases of camps. By a long and laborious train of chemical research, associated with attentive clinical observation, the doctor succeeded in tracing a connection between mountain fever — which is essentially a malarious fever — and an excess of "organic ammonia" in the water used by the patient. This "organic ammonia" is of course, in the trackless wildernes of the West, derived from the decomposition of vegetable matter. Washed from the air by rain and snow, even the mountain snow-water yields an appreciable quantity of the impurity. It is this water and the resulting spring-water, which causes the fever, "an adynamic remittant, for which the writer has suggested the name of *aquamalarial* fever." Thus far the author's discussion of malaria is quite unobjectionable, but in a subsequent portion of his essay he resumes the subject (pp. 145, 147, Vol. II.) and discourses of malarious germs in a manner that fills the thoughtful reader with astonishment. The doctor presents, as illustrations of diseases caused by air-borne vegetable spores, autumnal catarrh and rhus-poisoning, while cholera and typhoid fever are connected with specific germs, and malarial fever is to be in like manner referred to the action of vegetable germs. He believes "that certain germs exist and multiply in the decomposing vegetation of the soil, * * * and that when these find their way into the system and there meet with the vegetable tissues susceptible of the stage of decomposition which is native to them, they increase and multiply, developing according to circumstances, the various grades of malarial disease. * * * It is only in the alimentary canal that the malarial germs can find that fermentable vegetable matter which is necessary to their development. And as this supply of vegetable matter, susceptible of a certain decomposition, is not continuous in the system but intermittent, we have a hint as to the origin of one of the singularities of malarial disease," etc., etc. (pp. 146–7, Vol. II).

The above quotation affords a fine example of the charmingly inconsequential logic which carries captive the devotees of the germ theory. Let us analyze a little. Hay fever and rhus-poisoning are caused by the action of vegetable spores; but the action of the spores in those diseases is upon the surface of the body, and is of a directly irritant character, producing local inflammations. Spores are very tangible things, and the spores of all plants are likewise tangible and traceable. If now malaria is caused by vegetable germs, those germs must be spores of some kind. But in malarious diseases we can discover no evidence of inflammatory action upon either the inner or the outer surfaces of the body. If the intermittent paroxysms of fever are due to intermittent growths of cryptogams in the intermittent supplies of vegetable matter which enter the alimentary canal, the paroxysms should occur as often as the meals—but they do not; nor is there a particle of evidence of any peculiar cryptogamic intestinal vegetation in malarial fevers. The microscope does not reveal the presence of any such parasites in the fluids or cellular structures of the body. As for invisible growths and invisible germs we have no right to assume their existence as long as other hypotheses, based on actual observation, are sufficient to cover the ground. Our author seems to admit that his malarial germs may contribute to the living processes of the leaves of plants. But vegetable cells do not feed on other living cells; they consume inorganic and dead organic matter, but they are not known to absorb living germs. If vegetable spores and germs are the cause of malarial fever, there is no reason why human beings should ever become acclimatized. Spores and seeds will grow wherever they find warmth and moisture, such as the liquids of the human body always afford. Accordingly, the victims of hay fever and rhus-poisoning always suffer with each recurring crop of spores. But when disease is caused by substances which are capable of mingling, in the form of liquid or gaseous non-living matter, with the plasma of the blood, and when such substances enter the living cells, those living structures at once experience a modification of nutrition. This modification of nutrition constitutes the disease. If the disease were caused by the presence of vast numbers of living parasites in the liquids of the body, competing with the native cells for place and for food, the characteristic phenomena should be those which belong to embolism and to starvation. But there is nothing of the kind. The phenomena are those which belong to alterations of nutrition causing alteration of the functions of the natural cells. To this kind of change all the symptoms of infective diseases can be referred. As for "germs" too small for detection, we can know nothing of their natural history. We can only infer their presence from the consequences which should follow their introduction into the system. Since those consequences are mere perversions of the natural functions of the living cells, and since those functions are dependent upon the quality of the nutriment which is supplied to the cells, and since that nutriment always reaches the cells of the body in the condition of non-living, unorganized matter, it follows that the infective matters which excite infective diseases must be in that condition. They cannot be germs, alive and capable of indefinite multiplication. The living cells of the body are the only germs in the case; those cells always exhibit a tendency to adapt themselves to changes of nutriment, so that after a time they may exhibit a certain degree of healthy function, even though the blood be still contaminated by malaria. This we call acclimatization.

In accordance with this hypothesis it becomes very easy to see how malarial diseases are so largely dependent for their evolution upon changes of temperature and moisture. The cells of the tissues may only be so modified by their malarious diet that they can still perform their ordinary functions until the additional disturbances implied by fatigue or the action of damp night air, are superadded, when their equilibrium is completely overthrown and the phenomena of active disease appear. Quinine and other anti-periodics then become useful, not because of "germicide" properties, but by reason of their favorable effect upon the nutrition and defecation of the cells which are the seats of disease.

With these criticisms we will pass by the remainder of Dr. Smart's valuable paper — valuable in spite of its defects — and will call the attention of the reader to an essay on the "Hygiene of the Naval Merchant Marine," by Dr. Thomas G. Turner, Medical Director, U. S. N. Having seen considerable of both branches of the service, we can testify to the accuracy of the author's observations. His paper calls attention to the great hardship and danger which is produced on shipboard by crowding, dampness, improper diet and foul air. It seems, indeed, as if the sailor were compelled to encounter everything that ignorance and despotism can devise for his harm; and all growing out of traditionary errors on the part of non-medical officers regarding the essentials that are requisite for health and discipline. Dr. Turner's essay is so good and leaves so little to be desired, that it is with a feeling of real regret that one finds its closing pages disgraced by a miserable fling at the clergy for their philosophical position regarding the social management of syphilis. The doctor is evidently very much under the influence of the bias of position, and, like too many other narrow specialists, has not yet learned to examine this subject with the eyes of a philosopher. The whole paragraph should be omitted in future editions of the book.

The most interesting essay in the entire work is the paper on the "Hygiene of Coal Mines," written by Henry C. Sheafer, Esq., "Coal Editor" of *The Miner's Journal*, Pottsville, Pa. The author places before his readers, in a series of pen-pictures, the life of the coal miner from his earliest childhood at the pit-mouth till, prematurely bent with age and breathless with asthma, he sinks into the grave. It is one of the most satisfactory sketches ever presented to the medical public, quite overshadowing the excellent supplement on the "Hygiene of Metal Mines," furnished by Rossiter W. Raymond, Ph. D., editor of the *Engineering and Mining Journal*, published in New York City.

Following these interesting papers is a contribution to the hackneyed themes of "Infant Mortality and Vital Statistics," from the pen of Dr. Thomas B. Curtis, of Boston. The wise conclusion to which the author finds himself carried, is that "the chief agencies at work to bring about the excessive mortality of infants are the ignorance and poverty which prevail so extensively among certain classes of all urban populations." For the relief of this, "reliance must be placed chiefly upon the philanthropic exertions of charitable associations organized for the express purpose of assisting parents to understand and carry out the approved rules of infantile hygiene." And yet, it is not probable that even in this way would it be possible to effect any abiding change in the average results, for, as De Candolle and others have long ago said, the favoring conditions that are furnished by the organization of public and private charity "enables the weaker individuals to get along in life and to marry, whence results the generation of a stock of congenitally feeble infants. * * * In a high state of civilization such as is attained in the great cities of the present age, conditions of life exist which actually reverse the ordinary processes of natural selection and bring about the survival of the (physically) unfitted. These are enabled to live, to marry and to procreate; but their offspring, inheriting feeble or diseased constitutions, are necessarily short-lived."

Dr. Curtis' paper is full of suggestive material; but, like the majority of such writings, it in many particulars fails to go to the bottom of things and does not bring to the light those broad generalizations which sanitarians so persistently avoid.

Mr. Stephen P. Sharples, of Boston, Mass., contributes a brief compendium of the methods to be employed in detection of the adulteration of food. His testimony indicates the extreme difficulty which attends the effort to suppress such adulterations by statute law and also serves to encourage the belief that, after all, we are not in such danger from adulterated food as the professional alarmists would have us believe. He admits that "there has been no definite line laid down between good and bad meat." In fact, were there no competition between rendering establishments or fertilizer factories, we should hear very little about the existence of "emaciated beef," etc. that is unfit for fo od Eighty per cent. of the meat sent to the London market is the subject of tubercular disease, and, "to exclude it from the market would leave London without a meat supply." (*Brit. Med. Jour.*, Oct. 25, 1879, p. 647). As for milk, it is well known that water and burnt sugar are the only dangerous substances that are intentionally added to the fluid. When the milkman sells his watered milk for two cents a quart, while the pure article costs five or six cents, he is only gratifying that instinct of human nature which found expression in the sage remark of the small boy, aged five, when his sister, aged three, undertook to remonstrate in an uproarious manner against the limited area of the pool of maple-syrup which had been deposited near the center of her plate by a careful parent. "Spread it, sister, spread it!" exclaimed the young philosopher, as he proceeded to apply a layer of sweetness to the entire upper surface of his own plate and to the greater part of his countenance as well. Children—and the majority of grown people are only children written large—like to get something bulky and showy when they invest their money. They would rather have a silver dollar than a gold dollar any time, even if it is really worth ten or fifteen cents less. Consequently, where it cannot be shown that the addition of water introduces anything poisonous into the milk, it is the wisest way to let the trade regulate itself in accordance with the natural law of supply and demand. Such methods, however, would not suit that fondness for the display of power which is the chief characteristic of the average official.

The important subject of "Public Nuisances" is treated by Roger S. Tracy, M.D., one of the sanitary inspectors of the New York board of health. His paper is written in a very temperate manner and is a valuable storehouse of observations regarding the annoyances incident to city life. Fortunately, while it appears to be impossible for any one to turn around in a crowd without shocking the delicate sensibilities of some one, the nuisances produced by the activities of a crowded population are less dangerous to health than might be supposed. "Impartial testimony, for example, goes to show that the disagreeable emanations from fat and bone boiling establishments are not injurious to health, but rather the contrary." (Vol. II, p. 381.) Again, "it might be presumed that substances collected in such a reckless and indiscriminate way as rags, would be a common means of propagating contagious disease, but I have never heard of or seen a case in which this occurred, nor have I been able to find any account of one in my reading." (p. 418.) Speaking of the various processes connected with the utilization of animal refuse, such as glue-making, bone-boiling, fertilizer-making, fat-boiling, etc., etc., the evidence is conclusively against the idea that the offensive odors emitted in these processes are deleterious to health and the subject may be dismissed with the remark of the author that contrary opinions "coming from people who are trying to suppress a nuisance are naturally liable to be exaggerated and must be taken with allowance." (p. 426.) The author does not attempt to explain the manner in which living beings become tolerant of the most varied forms of discomfort; he merely enumerates the different nuisances and states their effect upon health. In fact, the adjustment of the human body to all kinds of noxious influences, so that they cease to operate injuriously, is a subject with which sanitarians seldom concern themselves.

"Quarantine" (with reference solely to sea-port towns) is ably discussed by the health officer of the port of New York, S. Oakley VanDerpoel, M.D., L.L.D. The subject possesses such interest and importance that we reserve the paper for a notice more extended than the limits of this review will permit. The secretary of the Louisiana State board of health contributes a disappointing essay on land quarantine, which seems to consist chiefly of a shallow argument in favor of throwing upon the national government the burdens which should be supported by State and civic organizations. The whole paper would do credit to its author as an argument addressed to the public through the periodical press; but in a volume like the present it is quite out of place.

Dr. Allan McLane Hamilton, of New York, the well-known author of a manual on nervous diseases, and Dr. Bachell E. Emmett have written the article on the "Management of Small-pox and other Contagious Diseases." Aside from considerable unnecessary descriptive matter pertaining to the diseases themselves, the essay is well written. It however, contains little that is new. In connection with the question of compulsory vaccination, the author very sensibly remarks, "Whatever may be the advantages of such a system abroad, it is certainly not suited to our own country. * * * It has been found, especially in New York, that an appeal to the common sense or fears of the public is sufficient, and that force is worse than useless. * * * Every year that passes shows an increased readiness upon the part of the lower classes to be vaccinated, and perhaps even in England the persuasive method would be the best, as it unquestionably is here." (p. 527). This is a principle which admits of almost indefinite extension in sanitary administration.

The "Hygiene of Syphilis" forms the subject of a brief and rather timid paper by Dr. F. R. Sturgis of New York. The author seems to be rather uncertain about the best course to pursue with reference to the suppression of syphilis, but he finally concludes that very little can be accomplished by the government "until a decided change takes place in the feeling and customs of the country." In other words, the matter is beyond the reach of all official intermeddling.

Dr. Elwyn Waller, chemist to the New York board of health, is the author of a valuable paper on Disinfectants, which may be considered the latest and perhaps the most authoritative utterance on the subject. It of course cannot be condensed for presentation in this review.

Dr. Tracy, the author of the paper on Public Nuisances, contributes another article entitled Village Sanitary Association. It has every where been found that, when the care of the health of the community has been abandoned to an official bureaucracy, matters soon relapse into their former condition. A general apathy and indifference settles down upon the authorities who have been specially charged with the work which properly belongs to every individual member of society, and the "public health" really deteriorates. Another evil, also, more frequently appears. The officials charged with the care of the "public health," find unnumbered opportunities for the initiation of costly public works — Fullerton avenue conduits, and their like — which are defended upon the ground of their alleged vital importance as means for the protection of the health of citizens. To save men from their saviors, and to diffuse real knowledge throughout the community, it is now found better to rely upon the independent voluntary efforts of citizens working together in the form of an association. This has been done with great advantage in several communities — notably in New Orleans during the past year. Dr. Tracy agreeably sketches the manner in which such associations may be formed, and the line of effort which they may pursue with advantage to the public.

The closing essay of the volume is written by Dr. D. F. Lincoln, of Boston. It consists chiefly of an application of the laws of hygiene to the individual care of children at school; and, though leaving scarcely anything to be desired, it calls for no special remark.

The unusual length of this review warns us against extended comment upon these volumes as a whole. They are not sufficiently condensed, and many subjects have really been considered by several of the essayists in succession. This was, however, inevitable, unless the editor had undertaken a closer revision than is usual under such circumstances. The work is a fine store-house of materials, gathered from the most varied sources; and will long remain the most convenient authority on all the subjects of which it treats. It is generally free from the extravagancies which characterize the writings of neophytes, though some of its contributors cannot always refrain from twanging the sanitary jews-harp. In spite of minor defects, the book will do a great deal of good, and should be carefully read by every physician. H. M. L.

ARTICLE X.—THE AMERICAN CYCLOPÆDIA OF DOMESTIC MEDICINE. Edited by S. P. Ford, M.D. 8 vo., 1215 pages. Chicago: E. P. Kingsley & Co., 1879.

This is an attempt to diffuse among the laity an acquaintance with the science of medicine and is the most successful effort of the kind we have seen.

The editor presents, in a popular manner, the principles that underlie the diagnosis and treatment of disease, and has given much space to hygiene. There seems to be a demand for such books, and while we may doubt the propriety of their publication, we must at least credit the editor of this with giving the people, along with much information, much sound advice. The tendency of the book will doubtless be to strengthen the regular profession and pull down the bulwarks of quackery. The necessity for a prompt appeal to the scientific physician and disastrous consequences that may follow the lead of the charlatan, are well stated on many pages. B.

